# Hock Lesions, Cow Hygiene, and Compost Quality in Compost-Bedded Pack Barns in Germany

**DOI:** 10.3390/ani15213205

**Published:** 2025-11-04

**Authors:** Phillip Andreas Guhl, Lisa Bachmann, Maike Heppelmann

**Affiliations:** 1 Clinic for Cattle, University of Veterinary Medicine Hannover, Foundation, 30173 Hannover, Germany; 2 Faculty of Agriculture and Food Sciences, University of Applied Science Neubrandenburg, 17033 Neubrandenburg, Germany; bachmann@hs-nb.de; 3 Institute for Farm Animal Biology (FBN), 18196 Dummerstorf, Germany

**Keywords:** compost-bedded pack barns, cow hygiene, hock lesions, compost quality

## Abstract

**Simple Summary:**

The prevalence of hock lesions and cow hygiene are indicators of housing system quality in dairy cattle. Compost-bedded pack barns (CBPs) have been shown to benefit the health and welfare of cows; however, further studies are needed to substantiate those findings. The present study evaluated various factors affecting cow hygiene and the prevalence of hock lesions in CBPs in southern Germany. The prevalence of hock lesions was 1.0% in the cold season and 3.9% in the warm season, which were lower than the results of previous studies in conventional housing systems. Cow hygiene was comparable to or better than that of conventional housing systems. Compost variables, compost quality, housing conditions and season affected cow hygiene.

**Abstract:**

The aim of the study was to evaluate the prevalence of hock lesions and cow hygiene as a cross-sectional study in dairy cows housed in compost-bedded pack barns (CBPs) in southern Germany. The effects of season, compost variables, and housing conditions on cow hygiene and hock lesion prevalences were also investigated. Eight farms that housed their cows in CBPs were visited once in the cold season and once in the warm season between January and December 2023. All cows (cold season n = 592; warm season n = 613) were scored for hygiene and hock lesions at each visit. Compost samples were collected for laboratory analysis, and the quality of the compost-bedded pack and condition of the lying surface and concrete walkways were assessed. The udder was the cleanest body zone in both seasons; poor udder hygiene (too dirty score) occurred in 15.0% of cows in the cold season and 7.5% in the warm season (*p* ≤ 0.05). Only 1% of the cows had a hairless area on a hock in the cold season compared with 3.8% in the warm season; 0.2% of the cows also had swelling of the hock in the warm season (*p* ≤ 0.05). The compost variables that impacted cow hygiene most frequently were dry matter and compost temperature. Based on our results, CBPs reduce the prevalence of hock lesions. Cow hygiene was affected by various factors, and therefore good management of CBPs is required for good cow hygiene.

## 1. Introduction

Housing is an important factor in dairy cow welfare. The type of housing plays a critical role in cow behaviour, comfort, and well-being [1,2,3]. Housing and barn design also impact the production performance of dairy cows [4,5]. Therefore, clean, comfortable housing is crucial for the health and longevity of cows [6]. According to the Welfare Quality^®^ assessment protocol for dairy cows, cow hygiene and the prevalence of hock lesions reflect the quality of a housing system and are used to evaluate the welfare principles of good housing and good health [7]. Free-stall barns have been the main housing system used for dairy cows in Germany, with cows mostly housed in cubicle barns [8]. Cubicle dimensions and bedding type affect animal welfare indicators, including cow cleanliness, skin lesions, and normal lying-down movement [9].

A high prevalence of hock lesions including hair loss, swelling, and wounds was reported in cows from 765 dairy farms in the north (N), east (E), and south (S) of Germany; 69.7% of cows in the N, 65.5% in the E, and 67.8% in the S had at least one lesion on one hock [8]. Further, the prevalence of hock lesions ranged from 22% to 68% in dairy herds in Austria and Germany; the prevalence was higher in conventional farms than in organic farms [10]. An American study found an association between housing type and severe hock lesions [11]. Studies have revealed certain issues with conventional free-stall barns: cows housed in restricted cubicles exhibit more atypical rising and lying motions, with lower duration and frequency of lying compared with cows in permissive cubicles [12]. More hock lesions were observed in cows housed in cubicle barns compared with straw yards [13]. A curb, soft lying surface, larger lying area, and more space under the cubicle partition were associated with a lower prevalence of hock lesions in cubicle barns [10].

Barn design and management also affect cow hygiene; poor barn cleanliness is associated with poor cow hygiene [1]. The lying area was the major factor affecting hygiene, which was associated with standing and lying behaviours, and the cleanliness of the environment [14]. In Germany, 22.6% (N), 18.4% (E), and 22.2% (S) of udder scores and 40.8% (N), 35.3% (E), and 32.7% (S) of leg scores were 3 or 4 (too dirty) [8]. In a Canadian study conducted in a free-stall barn, a cleanliness score of 3 or 4 (too dirty) was assigned to the flanks and upper legs of 74.1% of the cows, the lower legs of 99.7%, and the udder in 64.5% [14].

Compost-bedded pack barns (CBPs) are welfare-friendly, providing good comfort levels and promoting optimal health for dairy cows [15,16]. Compost-bedded pack barns have a concrete feed alley and an unobstructed bedded pack resting area. The bedding consists of organic material, such as sawdust or dry fine wood shavings, which decomposes under aerobic conditions. The bedded pack is tilled twice daily to provide a fresh surface and promote aeration [17]. The unrestricted open bedded pack allows cows to lie in a natural position; the head can be up or back, and cows can lie in lateral recumbency with the head on the ground [18]. Cows housed in CBPs had more lying time per day compared with other types of free-stall barns [19,20]. Improved comfort and animal welfare likely increase the longevity of cows housed in CBPs, resulting in higher parities compared with other types of free-stall barns [21]. A reduced culling rate was reported for one herd after conversion of the housing system to a CBP [22]. Improved animal health, such as a decrease in the somatic cell count and a reduction in mastitis infection rates, has also been described [22,23]. Compost-bedded pack barns have been associated with low prevalences of hock lesions [20,22,24,25] and lameness [24,25], and with good claw health with low prevalences of digital dermatitis, heel horn erosion, white line disease, ulcers, and interdigital hyperplasia [26,27]. Studies have shown better [28], similar [25,29], or worse [24] hygiene for cows housed in CBPs compared with other types of free-stall barns. Hygiene was affected by moisture, ambient temperature [23], and pack temperature [30].

The aim of this study was to evaluate cow hygiene and the prevalence of hock lesions in cows housed in CBPs in Southern Germany. Furthermore, we hypothesised that compost quality and season affect cow hygiene and hock lesions.

## 2. Materials and Methods

### 2.1. Selection of Herds

Thirty dairy herds that housed their cows in CBPs were contacted. For inclusion of herds in the study, all cows had to be housed in a CBP for a minimum of two years and have had no access to pasture. In addition, claw trimming had to be done at least once a year; foot and claw disorders were recorded as part of another study [27]. A total of eight herds located in southern Germany met the requirements.

### 2.2. Herd Visits

The herd visits took place between January 2023 and December 2023, with all data collected by one observer. Each herd was visited twice: once in the cold season (December to April) and once in the warm season (June to September).

A questionnaire covering general herd data, housing, compost management, and farmers’ opinions on certain aspects was completed with the farmer during the first visit. At both visits, the barn construction characteristics, housing conditions, and compost quality were assessed, and compost samples were collected for laboratory analysis. All cows were scored for hygiene and hock lesions.

Ear tag number, breed, age, parity, and days in milk were provided for all cows via access to HI-Tier (Traceability and information system of animals [Herkunftssicherungs- und Informationssystem für Tiere, Bayerisches Staatsministerium für Ernährung, Landwirtschaft, Forsten und Tourismus, Munich, Germany]).

### 2.3. Recording of Barn Conditions: Construction, Housing Conditions, and Management

The barn construction data included barn design, barn dimensions, lying area, concrete walkway, and barn equipment. All dimensions were measured with a laser measuring device. Barn equipment, such as fans, evaporative cooling systems, and scratching brushes, and the frequency of, and technology used for, concrete walkway cleaning were noted. The cleanliness, slip resistance, and condition of the lying area and concrete walkways were used to assess housing conditions. In addition, the number of cow pats in a 10 m^2^ area in the centre of the lying surface was counted. The cleanliness of concrete floors was assessed using a 4-point scoring system: 1 = clean floor with single cow pats, 2 = less than 50% of the area is soiled, 3 = more than 50% is soiled, and 4 = the entire area is covered in faeces [8]. A 3-point scoring system was used to record liquid accumulation on the concrete floor: 1 = no liquid accumulation, 2 = single puddles of liquid, and 3 = more than 50% of the surface is covered in liquid [8]. Two scoring systems were used to evaluate the condition of the lying surface and concrete walkway as good, moderate, or poor [27]. A commercially available weather station was used to determine air temperature [°C] and air humidity [%].

### 2.4. Scoring of Hygiene and Hock Lesions

All cows were scored twice by the same observer. A 4-point scoring system was used to assess the cleanliness of the udder, lower legs, and upper legs and flanks: 1 = clean, little or no evidence of faeces, 2 = clean, minor splashing of faecal material, 3 = distinct demarcated plaques of faecal material, and 4 = marked contamination, confluent plaques of faecal material, with scores of 3 and 4 defined as too dirty [6]. The lower region of the forelimbs was also scored. Hock lesions were scored using a 4-point scoring system: 0 = no hair loss or swelling, 1 = hair loss, no swelling, 2 = swelling, and 3 = severe swelling >7.4 cm, with possible purulent material, haemorrhage, or extensive dimensions [31].

The mean lying space available to each cow across all 8 herds was calculated and compared between cows with poor front leg, hind leg, and flank cleanliness for the cold and warm seasons.

### 2.5. Evaluation and Sampling of Compost Material

The colour and odour of the compost were used to evaluate its quality, and semi-quantitative testing was done to determine compost moisture. The “ball test”, “urine test”, and “boot test”, described in a previous study, were used to assess the moisture content of the compost [27]. The compost surface was assessed for shine or dullness, and the passages to the walkway were compared with the remaining lying surface. The texture of the compost was evaluated haptically. These sensory and semi-quantitative results were then incorporated into a previously used scoring system that considered all the results. Each farm was then assigned a good, moderate, or poor compost quality depending on its score [27].

To collect quantitative data, the compost temperature [°C] was measured using a GT1 temperature measuring probe (PFEUFFER GMBH, Kitzigen, Germany) at a depth of 20 cm, which is approximately the centre of the compost mattress. An AM 40 portable oxygen meter (Xylem Analytics Germany Sales GmbH & Co. KG, Weilheim, Germany) was used in combination with a soil probe to measure the oxygen saturation index [%]. The bedded pack resting area was divided into nine squares, and measurements were made in the centre of each square, as described in a previous study [29]. A compost sample of approximately 1 kg was taken from the area of each measurement point, hermetically sealed, and frozen for further analysis.

### 2.6. Laboratory Analysis of Compost Samples

The frozen compost samples were analysed for dry matter (DM) [%], carbon-nitrogen ratio (C:N), and pH at the Institute for Animal Nutrition, University of Veterinary Medicine Hannover (Germany).

To determine DM [%], a sample of compost was weighed in a dry aluminium dish and then dried at 103 °C in a drying oven until a constant weight was achieved. The DM [%] was calculated by determining the quotient of the weight out [g] and the weight in [g] and multiplying the result by 100.

The samples were ground in a centrifugal mill (ZM 200, Retsch GmbH, Haan, Germany) to determine pH and C:N. A 30–50 g sample of the ground compost was covered with distilled water. After 30 min, the pH value was measured using a pH combination electrode. To analyse carbon content, 300 mg of the ground sample was placed in a ceramic crucible and mixed with the same amount of tungsten (IV) oxide as a sample additive. The sample was then burned in the analyser (vario MAX cube, Elementar Analysensysteme GmbH, Langenselbold, Germany), and the carbon content determined using a thermal conductivity detector. 300 mg of the ground sample was burned in the analyser (vario MAX cube, Elementar Analysensysteme GmbH, Langenselbold, Germany), and the resulting molecular nitrogen was detected by a built-in thermal conductivity detector. A suitable software converted the results of the detectors to the C:N.

### 2.7. Statistical Analysis

Data were collated in Microsoft Excel (Microsoft Corporation, Redmond, WA, USA), and the SAS Enterprise Guide 7.1 (SAS Institute Inc., Cary, NC, USA) was used for further statistical analysis. The Shapiro-Wilk test was used to test for normality of the distribution of all quantitative variables. The mean ± standard deviation (SD) was calculated for normally distributed variables, and the median ± median absolute deviation values (MAD) for non-normally distributed variables. To compare the ordinal scores of hygiene, hock lesions, and compost quality between the cold and warm seasons, the Wilcoxon signed-rank test was used. Multiple logistic regression was done to rate the effect of compost quality, lying surface condition, and concrete walkway condition on hygiene for both seasons with calculation of the odds ratios and confidence intervals. A paired t-test was used to compare the seasonal results of the on-farm compost measurements and the laboratory analysis results. The Pearson correlation coefficient for normally distributed data and the Spearman correlation coefficient for non-normally distributed data were calculated to investigate the relationships among individual compost variables in both seasons. A Wilcoxon Rank Sum test was used to evaluate the effect of compost variables on cow cleanliness. The results of the PraeRi study [8] were compared with the prevalences of lower leg, flank, and udder cleanliness; this was achieved with a one-sample t-test for normal data. Differences were considered significant at *p* ≤ 0.05.

## 3. Results

### 3.1. Farm Structure

The business focus of all eight herds was dairy farming. Each herd had 64.5 ± 8.5 (median ± MAD) lactating cows housed in a CBP. A mean of 10.8 ± 4.6 dry cows were housed in a CBP at four farms, a free-stall cubicle barn at three farms, and a deep-bedded straw barn at one farm. A mean of 87.8 ± 38.6 young stock were housed in a free-stall cubicle barn at seven farms and a deep-bedded straw barn at one farm. German Fleckvieh was the dominant breed (82.2%), followed by Holstein Friesian (13.4%), and other breeds or crossbred cows (4.4%). The mean milk production per cow was 8417.4 ± 879.7 kg, and the average lactation number was 2 ± 1 (median ± MAD). A mean of 29.5 ± 0.4% of cows were in the first lactation, 26.5 ± 1.9% in the second, 18.5 ± 1.0% in the third, and 25.5 ± 2.5% in the fourth or higher lactation.

### 3.2. Barn Design, Barn Facilities, and Conditions

The main reason farmers converted their barns to CBPs was to improve animal welfare and health. The CBPs were built with a concrete walkway and an open, bedded pack resting area. Concrete walkways were cleaned with a manure removal system 14.5 ± 7.1 (median ± MAD) times a day in seven barns and a manure-collecting robot in one CBP. Seven CPBs had open fronts with cross ventilation and blinds. One CBP was obstructed by adjoining buildings that interfered with the ventilation. One CBP was built with large windows for ventilation.

The total bedded pack resting area comprised 625 ± 103.5 m^2^ (median ± MAD) with a planned space per cow of 10.8 ± 1.6 m^2^ (mean ± SD). The actual space per cow was 10.1 ± 1.1 m^2^ (median ± MAD) in the cold season and 9.9 ± 1.2 m^2^ (median ± MAD) in the warm season.

The condition of the lying surface had a score of 3/3 ± 0 points (median ± MAD), and the concrete walkway condition 5/5 ± 0 points (median ± MAD) in the cold and warm seasons.

The air temperature was lower in the cold season at 7.8 ± 2.9 °C (mean ± SD) than in the warm season at 24.7 ± 3.6 °C (mean ± SD; *p* = 0.0001). The air humidity in the cold season was 51.6 ± 14.0% (mean ± SD) and in the warm season 51.9 ± 11.1% (mean ± SD; *p* = 0.9742).

### 3.3. Compost

#### 3.3.1. Management

Sawdust, fine dry sieved wood shavings, and fine dry wood shavings were the most common bedding materials used. Some farmers also added small amounts of rape straw, spelt hulls, miscanthus, or horse dung. The bedded pack was tilled twice daily (2 ± 0 [median ± MAD]) using a cultivator on five farms and a rototiller on three farms.

Farmers added 17.5 ± 5 m^3^ (median ± MAD) fresh material to the bedded pack every 7 ± 1.5 days (median ± MAD) in the cold season and 21.1 ± 17.8 m^3^ (mean ± SD) fresh material every 19.8 ± 19.2 days (mean ± SD) in the warm season.

#### 3.3.2. Compost Quality

The compost quality, based on the sensory score, was 7.25/9 ± 1.5 points (median ± MAD) in the cold season and 7.75/9 ± 0.75 points (median ± MAD; *p* > 0.05) in the warm season. In the cold season, the compost quality was good on five farms and moderate on three farms. In the warm season, the compost quality was good on six farms, moderate on one, and poor on one. The results of on-farm measurements and laboratory analyses of compost samples in both seasons are presented in Table 1.

Correlations were calculated for pH, C:N, oxygen saturation index, compost temperature, DM, and air temperature. In the cold season, DM correlated with the compost temperature (0.89; *p* = 0.0030) and C:N with the oxygen saturation index (0.87; *p* = 0.0050). In the warm season, DM correlated with C:N (−0.94; *p* = 0.0004) and with the compost temperature (0.94; *p* = 0.0005). C:N correlated with the compost temperature (−0.91; *p* = 0.0017) in the warm season. No other correlations were found.

### 3.4. Scoring

#### 3.4.1. Hygiene Scoring

**Figure 1:**
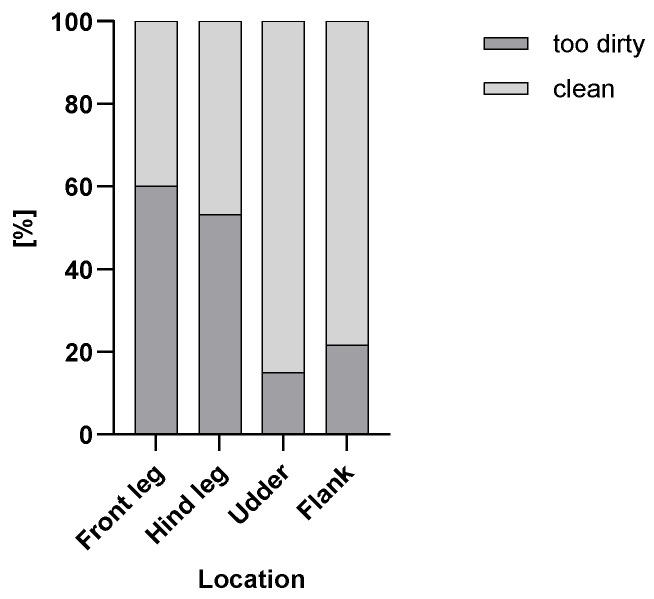
Percentage of too dirty and clean scored cows (n = 592) for the front legs, hind legs, udders and flanks in the cold season.

**Figure 2:**
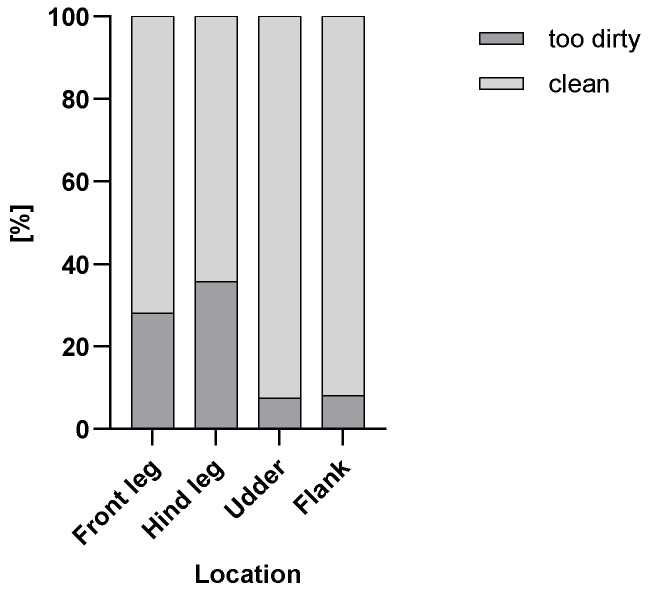
Percentage of too dirty and clean scored cows (n = 613) for the front legs, hind legs, udders and flanks in the warm season.

Figure 1 and Figure 2 show the percentages of too dirty and clean body parts of the scored cows in the cold (Figure 1) and in the warm (Figure 2) seasons.

The cows were cleaner in the warm season than in the cold season (*p* = 0.001).

**Figure 3:**
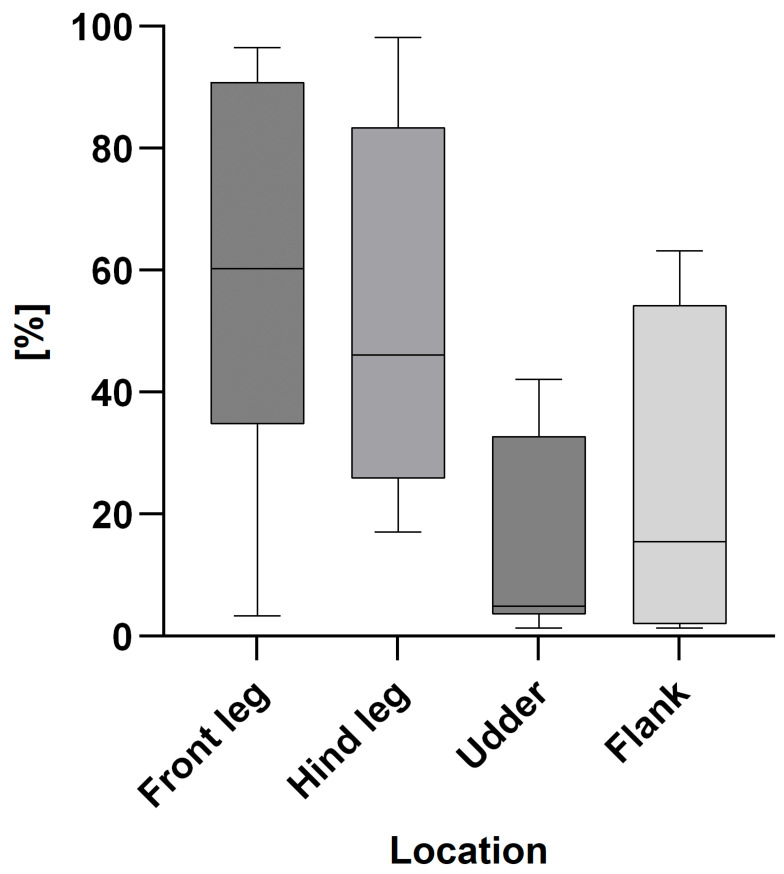
Percentage of too dirty scored cows on farm level (n = 8) for the front legs, hind legs, udders and flanks in the cold season expressed as box plots: median; Q 5/95.

**Figure 4:**
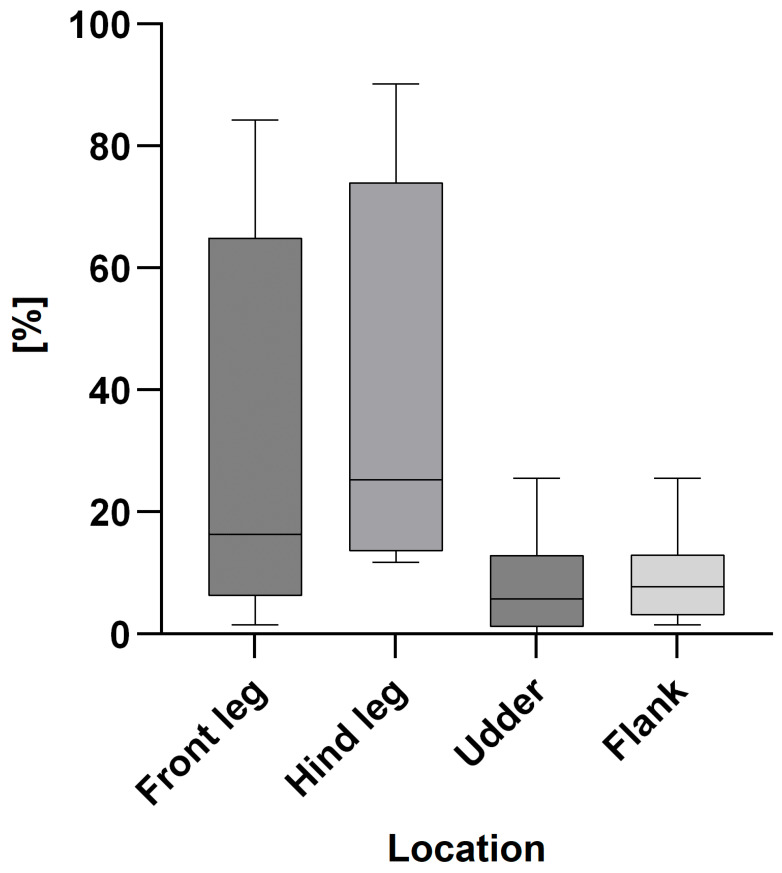
Percentage of too dirty scored cows on farm level (n = 8) for the front legs, hind legs, udders and flanks in the warm season expressed as box plots: median; Q 5/95.

Figure 3 and Figure 4 show the percentages of too dirty scored cows in the cold (Figure 3) and the warm (Figure 4) seasons on farm level.

In the cold season, hind leg hygiene did not differ from controls in conventional tie-stall or free-stall barns (32.7%; *p* = 0.1105) [8], and in the warm season, the prevalence of udders with a score of 3 or 4 (too dirty) was lower than the controls (18%; *p* ≤ 0.015) [8]. The results of the compost variables for cows that scored clean and too dirty are presented in Table 2 (cold season) and Table 3 (warm season). In the cold season, the lying space available to each cow was larger for cows with an udder score of 3 or 4 (too dirty) than for cows with a clean udder (11.3 ± 0.4 versus 9.5 ± 1.6 m^2^, *p* = 0.0086). In the warm season, cows with clean front legs (9.9 ± 1.4 versus 8.5 ± 0.6 m^2^; *p* < 0.0001), and clean hind legs (9.9 ± 1.4 versus 10.0 ± 0.2 m^2^; *p* = 0.0015) had a larger available lying area than cows with a score of 3 or 4 (too dirty).

#### 3.4.2. Hock Lesion Scoring

At the cow level, 99.0% of cows had no hock lesions in the cold season (n = 592) compared with 96.1% (n = 613; *p* = 0.0075) in the warm season. Hock lesion scores 2 and 3 were not seen in the cold season. In the cold season 1.0% of the cows had a hairless area. In the warm season, 3.8% of the cows had a hairless area, and 0.2% had hock swelling. At herd level, hock lesions were not seen in any of the cows (100.0 ± 0.0%, median ± MAD) in the cold season and in 97.9 ± 1.2% (median ± MAD) in the warm season. At the herd level, hairless areas did not occur in any of the cows (0.0 ± 0.0%, median ± MAD) in the cold season but in 2.1 ± 1.2% (median ± MAD) of the cows in the warm season. At the herd level, no cows had a hock lesion score of 2.

#### 3.4.3. Effect of Compost Quality, Lying Surface Condition, and Concrete Walkway Condition on Hygiene

Table 4 shows the results of the effect of different compost quality condition scores on hygiene for both seasons.

The quality of the lying surface had only an effect on the flank hygiene in the cold season (*p* < 0.0001; OR: 8.7; 95% Wald confidence interval: 2.0–38.2). The concrete walkway condition had no effect on cow hygiene in both seasons.

Due to the small number of cows with hock lesions the effect of compost quality, lying surface condition, and concrete walkway condition was not calculated.

## 4. Discussion

General farm data, barn design, barn conditions, including equipment, and management of the compost-bedded pack were discussed in a previous study and will not be covered again [27].

Composting is the biodegradation of organic materials by microorganisms under aerobic conditions [32]. The benefit of CBPs is based on these processes; composting generates heat, carbon dioxide (CO_2_), and water, which evaporates [32]. This occurs under a wide range of conditions and is affected by various factors, such as the C:N ratio, pH, DM, temperature, and oxygen concentration of the pack. Good composting requires an oxygen concentration of more than 5%, which corresponds to approximately 23.8% of the oxygen saturation index, a pH of 5.5–9, a moisture content of 40–65%, a C:N ratio of 20–40:1, and a temperature of 43.3–65.5 °C [32]. Microorganisms consume oxygen during the degradation of organic material, producing heat. A sufficient oxygen concentration is essential for good composting; otherwise, the process becomes anaerobic and less efficient [32]. Microorganisms use carbon for growth and energy and nitrogen for protein and reproduction, making these two elements essential for ideal composting. While pH does not appreciably affect composting, values above 8.5 encourage the conversion of nitrogen to ammonia, causing nitrogen loss [32]. Moisture is essential for chemical reactions, nutrient transport, and the movement of microorganisms. Low moisture levels are unfavourable because of slow microbial activity, while high moisture levels displace air in the bedding material, which can lead to anaerobic conditions [32]. High temperatures develop during microbial decomposition and dissipate via evaporation, air movement, and cold weather [32].

Sensory and semi-quantitative testing of the bedded pack in the eight CBPs yielded good results in the cold (7.25/9) and warm (7.75/9) seasons and was discussed in a previous study [27]. In the present study, the pH (cold season: 8.7; warm season: 9.1) and C:N ratio (cold season: 32.2; warm season: 26.8) were within the optimal range, and the oxygen saturation index (cold season 49.9%; warm season 23.9%) was above the minimum value of composting recommendations. Other studies of CBPs reported similar pH values (8.3 to 9.0) [17,33,34]. Our C:N ratio was higher compared with other studies, which recorded values between 2.4 and 17.6 [17,28,33]. The C:N ratio in two US studies was comparable to that in our study with 26.7 to 28.5 [23,35]. A high C:N ratio may have been attributable to fresh material being added to the bedded pack more frequently, and the availability of bedding material [28]. The oxygen saturation index was higher in the cold season (49.9%) than in the warm season (23.8%) and also higher than the recommended minimum value [32]. The results indicated good aeration of the bedded pack via the tilling procedure. However, the SD was larger in the cold season (22.8%), and thus, the measurements may not have been optimal, possibly associated with the measuring procedure. The compost was more humid in the cold season; when the soil probe was pushed into the compost, a channel may have been created, allowing air in and promoting spuriously high oxygen saturation indices.

The compost temperature was lower than the recommended values in the cold (25.8 °C) and warm (39.7 °C) seasons. Other studies reported temperatures of 35.9 °C to 47.2 °C [23,33,34,36], which were comparable to our results in the warm season. The DM in the present study was 39.1% in the warm season and lower as recommended in the cold season (33.6%). The moisture content was in agreement with the results of another study (63.4%) for both seasons [17]. Other reported moisture content values were similar to ours in the warm season, ranging from 56.1% to 59.9% [23,36]. Our lower pack temperature and DM in the cold season may have been attributable to external factors. The lower ambient temperature in the cold season leads to higher heat loss from the pack, cooling its surface and causing the pack temperature to approach that of the ambient air. Furthermore, the lower pack temperature results in lower water evaporation and a subsequent decrease in DM [23,32]. Other reasons for the lower temperature may have been reduced aeration of the bedded pack or the higher moisture content; both reduce the oxygen content of the bedded pack, thus decreasing microbial activity and heat production [32]. Two Brazilian studies reported moisture levels of 44.2 ± 8.3% [33] and 36.9 ± 5.2% [34], which were lower than those of the present study. This may have been due to higher barn temperatures, leading to water loss through evaporation. High barn temperatures reduce compost moisture [36]. In addition, the recommended threshold values did not apply specifically to CBPs, but instead to general composting on farms [32]. Proper composting in CBPs is a continuous process with frequent additions of fresh material. Overall, the quality of the bedded pack was good in both seasons based on sensory scores and laboratory analyses, despite minor deviations from the recommended values. Our results reflect good management routines.

Seasonal differences were measured for the pack temperature and the oxygen saturation index. The differences in compost temperature may have been attributable to external factors, as discussed previously. The differences in oxygen saturation index may have been due to the addition of fresh material. During the cold season, fresh material was added more often, perhaps providing better structure and porosity and improving aeration and composting. Fresh material was added less frequently in the warm season, which may have resulted in a more composted and a more compact pack, lowering its porosity [32]. To minimise the impact of different climatic conditions, farmers have to adjust their management depending on the climatic conditions of the season. In the cold season, possible adjustments include adding fresh materials more frequently, using bedding materials with small particle sizes thar compost quickly, providing more space per cow, and tilling the bedded pack more frequently, to support and improve the composting process. Conversely, in the warm season, farmers have to add new bedding material less frequently because the pressure of the climatic conditions is lower.

The positive correlation between DM and compost temperature in the cold and warm seasons was attributable to increased water evaporation at higher temperatures, leading to a higher DM [32,36]. Adding fresh bedding material more frequently in the cold season may explain the positive correlation between C:N and the oxygen saturation index. Fresh material has a higher C:N ratio than composted material. Compared to compost, fresh undecomposed material has more structure and better oxygen porosity, maintaining a high oxygen saturation index [32]. The negative correlation between the C:N ratio and the temperature may have been the result of the composting process; microorganisms use carbon, decreasing the C:N ratio, producing heat, and increasing the compost temperature [32] and DM [32]. The composting process may also explain the negative correlation between DM and the C:N ratio, as carbon decreases during composting and DM increases with the generation of heat [32].

The percentage of cows with poor hygiene is high in conventional farms. In free-stall barns, poor hygiene was reported for the upper legs and flanks in 19 to 71.9% of cows, the lower legs in 55 to 99.7%, and the udder in 19 to 65.5% [6,14,37]. In southern Germany, poor hygiene of the lower legs was seen in 32.7% of the cows and the udder in 22.2% [8]. To the authors’ knowledge, no studies have evaluated front leg hygiene; thus, it was included in the present study. We reasoned that the front legs are also affected by lying surface hygiene of the compost mattress and therefore, are just as dirty as the hind legs. It was not surprising that poorer front leg hygiene occurred in 58.0 ± 32.0% of the cows in the cold season than with 16.4 ± 12.6% in the warm season. These results were similar to those of hind leg hygiene with 51.9 ± 29.9% of cows affected in the cold season and 25.3 ± 12.6% in the warm season. The percentage of cows with poor hind leg hygiene in the cold and warm seasons was also lower or comparable to previous study results [6,8,14,37]. Poorer flank hygiene occurred in 24.5 ± 25.8% of the cows in the cold season than with 9.1 ± 7.8% in the warm season. The latter results were lower or comparable to the results of previous studies in free-stall and tie-stall barns [6,14,37]. The percentage of udders with a score of 3 or 4 (too dirty) was 5.0 ± 2.6% (median ± MAD) in the cold season and 8.4 ± 8.5% (mean ± SD) in the warm season, lower than the results of other studies [6,8,14,37]. Only the results of hind leg hygiene in the cold season and udder hygiene in the warm season could be compared statistically with reference values from southern Germany [8] because only these results were normally distributed. Hind leg hygiene did not differ between conventional housing systems and CBPs in the cold season. However, udder hygiene was better in CBPs than in conventional housing in the warm season.

Many factors affect cow hygiene, but in general, dirty barns lead to poor hygiene in cows [1,6]. In conventional housing systems, the lying area is a primary factor affecting hygiene; it is where cows are in contact with moisture and manure [14]. Thus, hygiene is affected by the cleanliness and dryness of the resting area [38]. This may explain the good upper leg and udder hygiene observed in our study. Providing good quality compost with a clean, dry lying surface prevents cows from excessive exposure to manure in both the cold and warm seasons. The lying area was just one section of the barn next to the concrete walkway, so cows were not exposed to all the manure produced. Concrete walkways affect lower leg hygiene when alley cleanliness is poor [6]. The frequency of alley scraping is associated with cow cleanliness, and alleys that are scraped more often result in better hygiene [14,39]. Longer standing times on concrete walkways were associated with poorer leg hygiene scores [1]. Lying time was longer for cows housed in CBPs compared with conventional free-stall barns [19], reducing the standing time on concrete walkways. Lower leg hygiene in CBPs not only depends on concrete walkways and their cleaning frequency, but also on compost quality. Depending on the moisture level of the compost, the lower legs may sink into the bedded pack and become soiled. Therefore, lower leg hygiene may be worse in the cold season because of higher moisture levels; the DM in the present study tended to be lower in the cold season than in the warm season. Good concrete walkway condition, together with good compost quality, resulted in lower leg hygiene scores that were similar to [6,8] or better than [14,37] scores in conventional housing systems. Our results are also in agreement with those of previous studies, in which cows in CBPs were cleaner [28], even though most studies reported that hygiene scores were similar to other housing systems [25,29].

The proportion of cows with a score of 3 or 4 (too dirty) was lower in the warm season than in the cold season for all four body zones. Lower ambient temperatures in the cold season likely lead to heat loss in the bedded pack and a decrease in water evaporation. The subsequent increase in moisture content likely facilitates the adherence of faecal and other material to the cows [23], promoting poor cow hygiene [36].

Compost DM and temperature had the most pronounced impact on cow cleanliness in both seasons. Cows with clean scores were generally housed in CBPs with higher compost temperatures and higher DM; these two variables were positively correlated. Higher compost temperatures promote water evaporation and a decrease in moisture [32,36], resulting in increased DM of the compost. Consequently, lower moisture levels are associated with better cow hygiene [36]. The effects of other variables on cow cleanliness (pH, oxygen saturation, C:N) were less consistent and therefore less important.

The prevalence of hock lesions in the present study was lower than in other studies. Only 1% of the cows had a hairless area in the cold season compared with 3.8% in the warm season. Additionally, only 0.2% of the cows had swollen hocks during the warm season. The higher prevalence of hock lesions in the warm season could be due to heat stress. In CBPs, a high stress condition based on climatic variables was reported [40]. Therefore, cows may avoid the bedded pack resting area and lie down on the concrete walkways instead. Studies reported a prevalence of 46 to 68% [3,10,41] of cows in conventional housing systems, and severe hock lesions were seen in 2.6 to 12.2% [11,41]. In southern Germany, the prevalence of hock lesions in conventional housing systems was 67.8%, and 15.6% had severe lesions [8]. One study of CBPs found a hock lesion prevalence of 0.5% [25], comparable to our results, while another had a prevalence of 24.1% for mild lesions and 1% for severe lesions in CBPs [22], which were higher than our findings. A high prevalence of hock lesions suggests compromised cow comfort [3]. Identified risk factors for hock lesions have been categorised as cow-related, management-related, or housing-related [42]; the farm system was considered a management-related risk factor [42]. Lower prevalences of hock lesions occurred in organic dairy farms compared with non-organic dairy farms [3,10], perhaps due to a longer grazing period [3]. Higher hock lesion prevalences were found in free-stall barns compared to straw yards [3,13,43], which supports the idea that hock lesions are associated with the quality and type of lying surface [11]. Soft lying surfaces reduced the prevalence of hock lesions compared with hard surfaces [10,44], and cows housed in deep-bedded cubicles had fewer hock lesions than cows housed on rubber mats [8,10,41]. Hock lesions are caused by abrasion, trauma, and pressure from hard surfaces [8,43]. The soft and elastic characteristics of bedding help prevent abrasions and allow sufficient blood circulation [10]. In barns with small cubicles or poorly designed barns, hock lesions can occur because cows have difficulty changing lying positions and rising and lying normally, and they may collide with barn fittings [10,43,45]. This is supported by the finding that fewer hock lesions were observed in cows with more space in cubicles [10]. Thus, the low prevalence of hock lesions in the present study was likely attributable to the soft, clean, dry bedded pack and the open, cubicle-free lying area, which prevented injuries from barn fittings.

Compost quality affected hygiene in both, the cold and warm seasons. Cows housed on compost with a higher compost quality score were cleaner, as cows housed on compost with a lower score. The scoring system is related to the compost DM, as the scoring system focused on moisture evaluation [27]. Compost of a lower compost quality score has higher moisture levels than compost with a higher score, which means faecal and other material adhere more easily to cows [23], resulting in poor hygiene scores [36]. The OR showed that the risk of poor hygiene was higher in cows housed on compost with a low compost quality score than compost with a high score.

Only in the cold season the lying surface condition had an influence on the flank hygiene, with cows having a higher risk for poor flank hygiene on a lying surface with a low lying surface condition score than with a high score. The lying surface score was based on cleanliness, slip resistance, and general subjective impression [27]. Therefore, the lying surfaces with a lower lying surface condition score, was dirtier than the lying surface of a higher score. The findings of a previous study suggested that dirty barns are the cause of dirty cows [1], which can explain the better flank hygiene of cows housed on lying surfaces with a high lying surface condition score in the cold season.

The Concrete walkways condition had no effect on the hygiene in both seasons. A possible reason for this was the cleanliness of the concrete walkways, reflecting good management practices. Another possible reason was reduced standing times on the concrete walkways because of longer lying times in CBPs [19].

## 5. Conclusions

Our results suggest that cows housed in CBPs have a lower prevalence of hock lesions than cows in conventional housing systems. Cow cleanliness is lower or comparable to conventional housing systems; however, cow hygiene is affected by various factors including season, compost variables, the quality of the compost-bedded pack, and the conditions of the lying surface and concrete walkway. Therefore, optimal management of the bedded pack is necessary to achieve good cow comfort in CBPs.

## Figures and Tables

**Table 1:** Results of on-farm measurements of the compost mattress and laboratory analysis of compost samples in the cold and warm seasons in eight compost-bedded pack barns.

	Season		
	Cold	Warm	*p*-Value
Temperature [°C]	25.8 ± 8.7 ^#^	39.7 ± 9.6 ^#^	0.0071
Oxygen saturation index [%]	49.9 ± 22.8 ^#^	23.9 ± 8.4 ^#^	0.0169
Dry matter [%]	33.6 ± 4.4 ^#^	39.1 ± 7.4 ^#^	0.0777
pH	8.7 ± 0.4 ^#^	9.1 ± 0.4 ^#^	0.0642
C:N	32.2 ± 12.3 ^#^	26.8 ± 5.5 ^#^	0.1875

^#^ Mean ± SD.

**Table 2:** Results of the compost variables (median ± MAD) for the hygiene scores clean and too dirty in the cold season.

		Front Leg Hygiene	Hind Leg Hygiene	Udder Hygiene	Flank Hygiene	
DM	Clean	342.7 ± 32.5 *	324.7 ± 18.0 *	324.7 ± 23.5 *	324.7 ± 23.5 *	
	too dirty	310.2 ± 9.0 *	310.2 ± 14.5 *	301.2 ± 9.0 *	310.2 ± 32.5 *	
pH	Clean	8.9 ± 0.2	8.9 ± 0.2	8.9 ± 0.0	8.9 ± 0.1	
	too dirty	8.9 ± 0.0	8.9 ± 0.0	8.9 ± 0.0	8.9 ± 0.0	
C:N	Clean	27.7 ± 5.2 *	27.7 ± 6.9	27.7 ± 5.2	24.4 ± 3.6 *	
	too dirty	24.4 ± 3.6 *	24.4 ± 3.6	24.4 ± 3.6	29.5 ± 5.1 *	
Compost temperature	Clean	27.8 ± 4.4 *	27.8 ± 4.4 *	23.4 ± 4.4 *	23.4 ± 4.4 *	
	too dirty	21.1 ± 3.9 *	21.1 ± 3.9 *	17.2 ± 1.9 *	21.1 ± 5.7 *	
Oxygen saturation index	Clean	39.8 ± 19.1 *	39.8 ± 22.0 *	47.2 ± 14.6 *	39.8 ± 19.1 *	
	too dirty	57.2 ± 10.0 *	58.9 ± 11.7 *	58.9 ± 1.7 *	58.1 ± 10.9 *	

* The median compost variable results differ between the clean and too dirty groups (*p* ≤ 0.05).

**Table 3:** Results of the compost variables (median ± MAD) for the scores clean and too dirty in the warm season.

		Front Leg Hygiene	Hind Leg Hygiene	Udder Hygiene	Flank Hygiene	
DM	Clean	443.4 ± 30.0 *	443.4 ± 30.0 *	426.8 ± 46.6	426.8 ± 46.6	
	too dirty	317.4 ± 22.6 *	411.0 ± 62.4 *	411.0 ± 62.4	364.2 ± 69.4	
pH	Clean	9.2 ± 0.2 *	9.3 ± 0.1 *	9.2 ± 0.2 *	9.2 ± 0.2 *	
	too dirty	9.0 ± 0.1 *	9.0 ± 0.1 *	9.1 ± 0.1 *	9.1 ± 0.1 *	
C:N	Clean	23.7 ± 4.6 *	23.3 ± 4.7 *	23.7 ± 5.1 *	23.7 ± 5.1 *	
	too dirty	32.4 ± 2.0 *	28.3 ± 4.6 *	28.3 ± 5.0 *	29.5 ± 4.9 *	
Compost temperature	Clean	44.0 ± 4.3 *	45.5 ± 2.8 *	44.0 ± 6.2 *	44.0 ± 6.2 *	
	too dirty	28.3 ± 3.8 *	43.0 ± 7.2 *	43.0 ± 7.2 *	38.2 ± 9.9 *	
Oxygen saturation index	Clean	19.5 ± 4.1 *	19.4 ± 3.9	19.5 ± 4.1	19.5 ± 4.1	
	too dirty	15.5 ± 0.1 *	19.5 ± 4.1	19.5 ± 4.1	19.5 ± 4.1	

* The median compost variable results differ between the groups clean and too dirty (*p* ≤ 0.05).

**Table 4:** Effect of compost quality on cow hygiene for both seasons.

	Cold Season			Warm Season		
	*p*-Values	OR	95% Wald Confidence Interval	*p*-Values	OR	95% Wald Confidence Interval
Front leg hygiene	<0.0001	39.6 *	12.9–121.3	<0.0001	32.0 ^#^	13.4–76.4
Hind leg hygiene	<0.0001	21.2 *	9.2–49.1	<0.0001	36.0 ^#^	13.3–97.5
Udder hygiene	<0.0001	11.1 *	3.3–37.6	0.0002	3.5 ^#^	1.5–7.9
Flank hygiene	<0.0001	8.7 *	2.0–38.2	0.0001	5.4 ^#^	2.2–13.2

* The odds ratio (OR) was specified for the worst (4.5) in relation to the best (9) compost quality score in the cold season. ^#^ The OR was specified for the worst (0) in relation to the best (8.5) compost quality score in the warm season.

## Data Availability

The original contributions presented in this study are included in the article. Further inquiries can be directed to the corresponding author.

## References

[1] Robles I., Zambelis A., Kelton D.F., Barkema H.W., Keefe G.P., Roy J.P., von Keyserlingk M.A.G., DeVries T.J. (2021). Associations of freestall design and cleanliness with cow lying behavior, hygiene, lameness, and risk of high somatic cell count. J. Dairy Sci..

[2] Hultgren J. Cattle welfare aspects of animal hygiene. Proceedings of the XIth International Congress in Animal Hygiene of the International Society for Animal Hygiene (ISAH).

[3] Rutherford K., Langford F., Jack M., Sherwood L., Lawrence A., Haskell M. (2008). Hock injury prevalence and associated risk factors on organic and nonorganic dairy farms in the United Kingdom. J. Dairy Sci..

[4] Næss G., Bøe K.E., Østerås O. (2011). Layouts for small freestall dairy barns: Effect on milk yield for cows in different parities. J. Dairy Sci..

[5] Thompson J.S., Hudson C.D., Huxley J.N., Kaler J., Robinson R.S., Woad K.J., Bollard N., Gibbons J., Green M.J. (2022). A randomised controlled trial to evaluate the impact of indoor living space on dairy cow production, reproduction and behaviour. Sci. Rep..

[6] Cook N.B. The Influence of Barn Design on Dairy Cow Hygiene, Lameness and Udder Health. Proceedings of the 35th Annual Convention of the American Association of Bovine Practitioners.

[7] WelfareQuality (2023). Welfare Quality Assessment Protocol for Dairy Cows. Version 3.0. Welfare Quality Network. https://www.welfarequalitynetwork.net/media/1319/dairy-cattle-protocol.pdf.

[8] PraeRi (2020). Animal health, hygiene and biosecurity in German dairy cow operations—A prevalence study (PraeRi). Final Report.

[9] Gieseke D., Lambertz C., Gauly M. (2020). Effects of cubicle characteristics on animal welfare indicators in dairy cattle. Animal.

[10] Brenninkmeyer C., Dippel S., Brinkmann J., March S., Winckler C., Knierim U. (2013). Hock lesion epidemiology in cubicle housed dairy cows across two breeds, farming systems and countries. Prev. Vet. Med..

[11] Adams A.E., Lombard J.E., Fossler C.P., Roman-Muniz I.N., Kopral C.A. (2017). Associations between housing and management practices and the prevalence of lameness, hock lesions, and thin cows on US dairy operations. J. Dairy Sci..

[12] Brouwers S.P., Simmler M., Scriba M.F., Savary P. (2024). Cubicle design and dairy cow rising and lying down behaviours in free-stalls with insufficient lunge space. Animal.

[13] Potterton S.L., Green M.J., Millar K.M., Brignell C.J., Harris J., Whay H.R., Huxley J.N. (2011). Prevalence and characterisation of, and producers’ attitudes towards, hock lesions in UK dairy cattle. Vet. Rec..

[14] DeVries T., Aarnoudse M., Barkema H., Leslie K., Von Keyserlingk M. (2012). Associations of dairy cow behavior, barn hygiene, cow hygiene, and risk of elevated somatic cell count. J. Dairy Sci..

[15] Pilatti J.A., Vieira F.M.C. (2017). Environment, behavior and welfare aspects of dairy cows reared in compost bedded pack barns system. J. Anim. Behav. Biomete.

[16] de Andrade Kogima P., Diesel T.A., Vieira F.M.C., Schogor A.L.B., Volpini A.A., Veloso G.J., Ferraz P.F.P., Zotti M. (2022). The Welfare of Dairy Cows in Pasture, Free Stall, and Compost Barn Management Systems in a Brazilian Subtropical Region. Animals.

[17] Janni K.A., Endres M.I., Reneau J.K., Schoper W.W. (2007). Compost dairy barn layout and management recommendations. Appl. Eng. Agric..

[18] Endres M.I., Barberg A.E. (2007). Behavior of dairy cows in an alternative bedded-pack housing system. J. Dairy Sci..

[19] Eckelkamp E., Gravatte C., Coombs C., Bewley J. (2014). Case study: Characterization of lying behavior in dairy cows transitioning from a freestall barn with pasture access to a compost bedded pack barn without pasture access. Prof. Anim. Sci..

[20] Fernandez A., Mainau E., Manteca X., Siurana A., Castillejos L. (2020). Impacts of compost bedded pack barns on the welfare and comfort of dairy cows. Animals.

[21] Leso L., Pellegrini P., Barbari M. (2019). Effect of two housing systems on performance and longevity of dairy cows in Northern Italy. Agron. Res..

[22] Barberg A.E., Endres M.I., Salfer J.A., Reneau J.K. (2007). Performance and welfare of dairy cows in an alternative housing system in Minnesota. J. Dairy Sci..

[23] Black R.A., Taraba J.L., Day G.B., Damasceno F.A., Bewley J.M. (2013). Compost bedded pack dairy barn management, performance, and producer satisfaction. J. Dairy Sci..

[24] Lobeck K.M., Endres M.I., Shane E.M., Godden S.M., Fetrow J. (2011). Animal welfare in cross-ventilated, compost-bedded pack, and naturally ventilated dairy barns in the upper Midwest. J. Dairy Sci..

[25] Costa J.H.C., Burnett T.A., von Keyserlingk M.A.G., Hotzel M.J. (2018). Prevalence of lameness and leg lesions of lactating dairy cows housed in southern Brazil: Effects of housing systems. J. Dairy Sci..

[26] Burgstaller J., Raith J., Kuchling S., Mandl V., Hund A., Kofler J. (2016). Claw health and prevalence of lameness in cows from compost bedded and cubicle freestall dairy barns in Austria. Vet. J..

[27] Guhl P.A., Steiner A., Bachmann L., Heppelmann M. (2025). The Effect of Compost-Bedded Pack Barns on Claw Health and Lameness in Dairy Herds in Southern Germany. Animals.

[28] Biasato I., D’Angelo A., Bertone R., Odore R., Bellino C. (2019). Compost bedded-pack barn as an alternative housing system for dairy cattle in Italy: Effects on animal health and welfare and milk and milk product quality. Ital. J. Anim. Sci..

[29] Eckelkamp E.A., Taraba J.L., Akers K.A., Harmon R.J., Bewley J.M. (2016). Sand bedded freestall and compost bedded pack effects on cow hygiene, locomotion, and mastitis indicators. Livest. Sci..

[30] Favero S., Portilho F.V.R., Oliveira A.C.R., Langoni H., Pantoja J.C.F. (2015). Factors associated with mastitis epidemiologic indexes, animal hygiene, and bulk milk bacterial concentrations in dairy herds housed on compost bedding. Livest. Sci..

[31] Fulwider W.K., Grandin T., Garrick D.J., Engle T.E., Lamm W.D., Dalsted N.L., Rollin B.E. (2007). Influence of free-stall base on tarsal joint lesions and hygiene in dairy cows. J. Dairy. Sci..

[32] Rynk R., Van de Kamp M., Willson G.B., Singley M.E., Richard T.L., Kolega J.J., Gouin F.R., Laliberty L., Kay D., Murphy D. (1992). On-Farm Composting Handbook (NRAES 54).

[33] Freu G., Garcia B.L.N., Tomazi T., Di Leo G.S., Gheller L.S., Bronzo V., Moroni P., Dos Santos M.V. (2023). Association between Mastitis Occurrence in Dairy Cows and Bedding Characteristics of Compost-Bedded Pack Barns. Pathogens.

[34] Oliveira V.C., Damasceno F.A., Oliveira C.E.A., Ferraz P.F.P., Ferraz G.A.S., Saraz J.A.O. (2019). Compost-bedded pack barns in the state of Minas Gerais: Architectural and technological characterization. Agron. Res..

[35] Damasceno F.A., Day G.B., Taraba J.L., Barbari M., Oliveira C.E.A., Frigeri K.D., Vieira F.M.C., Bambi G. (2022). Determination of Thermal, Chemical and Physical Properties of Bedding Materials for Compost Dairy Barns. Animals.

[36] Eckelkamp E.A., Taraba J.L., Akers K.A., Harmon R.J., Bewley J.M. (2016). Understanding compost bedded pack barns: Interactions among environmental factors, bedding characteristics, and udder health. Livest. Sci..

[37] Robles I., Kelton D.F., Barkema H.W., Keefe G.P., Roy J.P., von Keyserlingk M.A.G., DeVries T.J. (2020). Bacterial concentrations in bedding and their association with dairy cow hygiene and milk quality. Animal.

[38] Andreasen S., Forkman B. (2012). The welfare of dairy cows is improved in relation to cleanliness and integument alterations on the hocks and lameness when sand is used as stall surface. J. Dairy Sci..

[39] Magnusson M., Herlin A., Ventorp M. (2008). Effect of alley floor cleanliness on free-stall and udder hygiene. J. Dairy Sci..

[40] Andrade R., Tinôco I., Damasceno F., Valente D., Oliveira C., Oliveira V., Rossi G., Barbari M. (2023). Analysis of environmental conditions in two different Compost Bedded Pack Barn systems for dairy cattle. Agron. Res..

[41] Cook N., Hess J., Foy M., Bennett T., Brotzman R. (2016). Management characteristics, lameness, and body injuries of dairy cattle housed in high-performance dairy herds in Wisconsin. J. Dairy Sci..

[42] Kester E., Holzhauer M., Frankena K. (2014). A descriptive review of the prevalence and risk factors of hock lesions in dairy cows. Vet. J..

[43] Haskell M., Rennie L., Bowell V., Bell M., Lawrence A. (2006). Housing system, milk production, and zero-grazing effects on lameness and leg injury in dairy cows. J. Dairy Sci..

[44] Kielland C., Ruud L., Zanella A., Østerås O. (2009). Prevalence and risk factors for skin lesions on legs of dairy cattle housed in freestalls in Norway. J. Dairy Sci..

[45] Potterton S., Green M., Harris J., Millar K., Whay H., Huxley J. (2011). Risk factors associated with hair loss, ulceration, and swelling at the hock in freestall-housed UK dairy herds. J. Dairy Sci..

